# Attitudes to Cryptocurrencies: A Comparative Study Between Sweden and Japan

**DOI:** 10.1007/s12626-021-00069-6

**Published:** 2021-03-17

**Authors:** Rickard Grassman, Vanessa Bracamonte, Matthew Davis, Maki Sato

**Affiliations:** 1 Stockholm University, Stockholm, Sweden; 2 KDDI Research, Inc., Fujimino, Japan; 3 Uppsala University, Uppsala, Sweden; 4 The University of Tokyo, Tokyo, Japan

**Keywords:** Cryptocurrencies, Bitcoin, Money, Attitudes, Autonomy

## Abstract

In this paper, we explore how cryptocurrencies have been received in Sweden and Japan, and what specific attitudes and discourses may reveal about the ethical implications surrounding this new technology. By way of topic modelling prevalent discourses on social media among users of cryptocurrencies, and teasing out the more culturally situated significance in such interactions through discourse analysis, our aim is to unpack the way certain tropes and traces around the notion of autonomy may provide a fruitful lens through which we may discern how this technology has been received in each respective country. The ultimate aim of the paper is to shed light on the attitudes that inform the way this technology is perceived and the cultural and ideological nuances that this brings to the fore, as well as how this culturally nuanced view may help us better discern the potential advantages and ethical challenges associated with this new technology.

## Introduction

On September 15th 2008, Lehman Brothers went bankrupt and sent financial markets into free fall, freezing up credit that even manufacturing industries rely on to operate. Global capitalism as we know it was in cardiac arrest, and the public outrage over the blatantly unethical credit schemes on a systemic-wide scale (CDOs and CDSs) resulting in trillions of taxpayer dollars spent on failing banks, has taken many forms. One initially rather discrete act of defiance comes through by way of an encrypted message to a small mailing-list of hackers calling themselves ‘*Cypherpunks*’ [30]. The mail was sent by someone under the pseudonym Satoshi Nakamoto, who made the audacious claim of having cracked the code that will free us from this current and failing banking system (Nakamoto 2009). Apart from Wikileaks and Julian Assange, the Cypherpunk movement had been as hidden from public view as the very combination of the *Cyberpunk* ethos in conjunction with a fetish for cryptography would see fit. In the ascending age increasingly marked by what Zuboff [32] will later refer to as one of surveillance capitalism, cryptography and cyberpunk is not as odd a pair as one might think. Much less for dwellers in the cyberpunk universe with striking embodiments from William Gibson’s modern classic *Neuromancer* to films like *Bladerunner* and *the Matrix*, in which it becomes increasingly apparent that escaping the gaze of an all-powerful AI assisted state and/or dominant corporate agendas essentially means to move out of sight. Not unlike a rigged game of oligopolistic finances that wins for the monied few and occasionally loses for all. Thus, the unknown voice under the pseudonym Satoshi Nakamoto must have laid out words as welcome as they were familiar to Cypherpunk and primary addressee Hal Finney, when allegedly having cracked the money code with the blockchain innovation associated with Bitcoin, Satoshi claimed to have created ‘the absolute autonomy of money’ [23].

What is so interesting about this new innovation is that despite its humble beginning it challenges the very core infrastructure of third-party trust that underpins the financial system itself, and more specifically the trust in the institutions of our capitalist economies that facilitate value transmissions to operate sustainably enough to function as money. At the heart of the problem that this new innovation brings to the fore lies the age old ethical question around autonomy and paternalism, or in other words, whether each of us are the better judges of what is good and worthwhile for our own physical or economic well-being (cf. [24]), or if that adjudication can be better handled by state institutions or other legitimate authority overriding the individual (cf. [26]).

In writing these words, the world is currently facing a Covid 19 pandemic in which the well-being of millions of people is at stake, yet it has invoked very different responses from one country to the other ranging from more paternalistic lockdowns and the sanctioning of deviancy, to less constraining recommendations as in the case of Sweden where the appropriate measures are ultimately still entrusted to individual informed consent and discretion. In the case of Japan on the other hand, the Covid 19 pandemic has among other things accelerated the discussion among government authorities to promote a shift to a more cashless society (sovereign virtual currency) instead of the more habitual use of ‘dirty’ cash that they fear may exacerbate viral contagion. This is by no way to equate the lethal pathogen of the pandemic with money or monetary innovations in any form, nor to make too much of the cleanliness aspect of certain types of money as opposed to others, whether physically or symbolically. Nevertheless, the way one country confronts a lethal pathogen or for that matter a disruptive innovation that threatens established institutions, may each in their own way reveal something about the culture through which one response or other is borne out. In a similar vein, we expect the global spread of cryptocurrencies and the blockchain technology that essentially enables money and monetary transactions to function in the absence of centralised authority such as governmental institutions in charge of monetary policy, to invoke quite different responses that reveal culturally significant tendencies around central tropes like that of ‘autonomy’ ranging from one culture to the other.

In this paper, we will be looking at Japan and Sweden and specifically how these cryptographic technology enabling peer-to-peer transactions without third-party mediation have been received in each respective country, which is likely to invoke a rich pallet of culturally contingent nuances as to the significance of autonomy, that this new technology brings to light. In other words, the empirical component of the paper explores how users in Japan and Sweden reason about this new technology, and in the light of each emergent culturally situated discourse on the subject matter, we may discern how different attitudes and behaviours reveal the cultural and ideological proclivities and tensions on the ethical meaning of autonomy in each country. Our aim is to illustrate some of the differences or similarities between Japan and Sweden regarding their respective use and the salient discourses around cryptocurrencies and the possibility of money without centralised authority or third-party control. Moreover, our ambition in so doing is to tease out what this may say about the broader ideological and cultural issues that we discern to be associated with such culturally specific nuances. A mixture of qualitative and quantitative methods will seek to identify and explore the separate cultural, institutional and historical contexts between empirical differences in the rate of adoption and use of virtual currencies among users, and the discourses under analysis here will help us accentuate the significance thereof.

Our analysis will bring to the fore the ways in which the particular ideological purpose of freeing us; ‘from our dependence on banks’ articulated by the unknown inventor Satoshi Nakamoto, have fared alongside the technology it has given rise to through the diverse socio-cultural landscapes that obviously distinguishes Japanese users from their counterparts in Sweden. To facilitate such analysis and to access the most fertile fora within which users interact and elaborate on the meaning of the technology, we have chosen discourse analysis of social media as the most suitable approach in this regard by way of enabling us to monitor how significances are understood, articulated and evolve over time. The more precise approach will be further explained in the methodology section, where we elaborate further on why we have chosen to conduct a Foucauldian inspired discourse analysis on social media to accentuate the power beyond individuals that such media along with his particular philosophy make strikingly clear. This analysis we argue, not only captures the power dynamic apparent in social media under analysis, which we also deem to be a more immediate and unfiltered representation of user attitudes than the editorialised narratives of established media. It also gives a more nuanced illustration of the relational aspect of power and how certain interactions of words and concepts will enact power in such exchanges, rather than through any one particular individual exerting it.

In shifting the analysis from the individual realm to that of a more relational understanding of power in discerning autonomy vis-à-vis this new technology, we will need to explore on the one hand how disciplining tropes and authorities are much more subtly embedded in discourse and constraining on autonomy in their own right, as are now a plethora of countering external institutions and powerful interests stressing how the other side of cryptocurrencies like Bitcoin and its empowering narratives, are laced with facilitating dealings in narcotics, tax evasion and other illicit activities including terrorism (Committee of Homeland Security and Governmental affairs, US Senate, 2014).

In sum, what we hope to do in this paper is not just foreground how these sets of ethical concerns and principles within and around the alleged autonomy in question, not only provides us with a set of applied ethics problems, albeit with extensive theoretical ramifications in its own right. In addition, we explore and problematize the ongoing power dynamic that brings together these ethical and ideological perspectives in the public discourse of social media to tease out the way in which this significance is created and evolve over time through the subtle power of this discursive dynamic, and how it may differ from one country to the next, and moreover, what such distinctions may say about the ethical and ideological leanings that Sweden and Japan bring to the fore in confronting this new technology and its accompanying promise of ‘autonomy’.

### The Ideological Blind Spot of Monetary History

The history of money is not as straightforward as it may appear, and most accounts in fact fail to see that there is a difference between a history of money and a history of coinage [11, 20]. It is perhaps understandable that this very technology of transferability and permanence mastered around 600 BCE in Lydia of Anatolia, as well as in the Ganges Valley and in city states around the Yellow river of what is now modern-day China, at about the same time [11], is when the evolution of money becomes increasingly visible to historians and archaeologists. Indeed, it was a tremendously significant development in its own right. However, it did not set off the story of money as we know it because money is not necessarily embodied in objects to be passed around to facilitate transactions, but had instead existed at least since the ancient Sumerians started inscribing precise debt relations among its citizens on clay tablets about 2500 BC. It is in fact here and not with the much later innovation of coined money that we start to see a system of inscribed debt relations that for the first time starts to function as money, as long as the integrity and incorruptibility of these tablets sufficed to sustain public trust therein.

In contrast, from the time of English philosopher John Locke onwards with notable accounts by classical economic scholars such as Adam Smith and John Stuart Mill, the story was one in which before money as exchangeable objects emerged (*qua* coins). According to this view societies and markets must have been characterised by the way armchair philosophers in monied economies tend to imagine ones without, which is essentially how the barter story comes about. This in spite of the fact that there is scarcely any empirical evidence of any barter societies ever existing, and in turn a great deal of evidence of various forms of credit arrangements preceding coinage [11]. Nevertheless, this narrative curiously still finds its way into any given Economics textbook of the more mainstream variety to this day.

Obviously, the story was a compelling one, albeit not so much for empirical reasons as for ideological ones. Indeed, for anyone hoping to provide a firm ideological grounding for the free market, not to mention founding the discipline of Economics in the case of Smith, the story worked hand in glove so to speak. Not only did it illustrate the natural inclinations of trucking and bartering as fundamental to the human condition, nor just showing how hopefully inconvenient these double-coincidences of demand must have been working before coined money came along as a technical fix. However, perhaps most importantly the story salvages this ideological view of history from having to grapple with the foundational role of state and central authority. Not to mention the inherent inequality of debt being endemic to the free market and not just the eventual outcome and modern financial innovation they much rather envision credit arrangements to be [11]. In other words, money is not necessarily the object of exchange but rather the precise social relation that is being quantifiably assessed through an impersonal system of value exchange, be it through inscription, possession of specific objects such as coins, paper bills or otherwise.

To this day, the ideological tension rages on between what we may want to place in two camps; the commodity theory variety following Adam Smith’s [28] assertion that money emerges naturally through the market mechanism and human nature, and the state or credit theory of money according to which money is created by a centralised authority such as in many cases a state that becomes the ultimate arbiter of each and all debt relations made up by the monetary activity. In retrospect, it appears that the state/credit theory has been more historically accurate in this debate all along considering the archaeological and anthropological insights we now have about Sumerian scriptures testifying to top–down central authority and debt relations, as opposed to barter and organically generated commodity money characterising early monetary systems. Moreover, debt relations imply power imbalances and centralised authority (or state function) to safeguard recordkeeping, not the most popular notions among free market enthusiasts, whereas the more ideologically resonant barter story imply natural human bartering inclinations creating markets and by extension money as stand in commodities to facilitate such transactions.

What the invention of coinage did do, however, even if not the creation of money per se as discussed above, was to greatly increase the autonomy of money as it depersonalised the power of money into the perceived value of the object/token/coin to be passed around. This we argue equally applies to the innovation of cryptocurrencies explored here, which we will unpack further in the following section as it relates to Sataoshi’s famous claims about creating ‘the absolute autonomy of money’, as well as the ethical implications associated therewith. To better grasp the essence of money, however, to then move on to discuss its implications for autonomy and ethics in the following section, the first step in the present section has been to tease out how its history has not been immune to the many ideologies that have endeavoured to try and account for its essence and characteristics in various ways.

Now, in spite of the justified critique of Smith’s idea regarding the historical accuracy of commodity money emerging organically through the market and the obvious ideological bias associated therewith, could we not perhaps read the ascent of cryptocurrencies as at least a partial vindication of Smith’s [28] idea albeit in retrospect? At least it is now clear that blockchain technology for one may supplant the need of a state for centralised top–down authority for something to function as money. What is more, cryptocurrencies bring to the fore this very ideological kernel that has been at the heart of this debate since Smith [28] came to formulate his position, and it is precisely by embodying the very proof of a decentralised form of money independent of any state or centralised monitoring authority, that it has such an appeal and why it pertains so strikingly to the question of autonomy in ethics.

#### Autonomy, Power and Discourse

Indeed, the concept of autonomy has a long historical etymology by which its meaning and first use is derived well back from the ancient Greeks discussing sovereign statehood and its constitutive ability to be a lawgiver unto oneself as a state (‘autos’—self, ‘nomia’—law). However, it is only with the ascent of the Enlightenment and most notably with Immanuel Kant that autonomy becomes a primary concern at the individual level, where as it were Kant’s view would envision individuals reaching maturity through assuming autonomy by way of reasoning for oneself rather than deferring authority onto tradition, state, church etc. Autonomy according to Kant refers to a moral agent’s ability to freely and rationally adopt moral policies and act accordingly [17], which holds a rather optimistic view of human faculties in the absence of circumstances constraining our freedoms. This may perhaps in some sense be true of Foucault as well, although in a less optimistic vein as the space of autonomy is diminishingly small if even at all possible considering the ubiquity of normative and disciplinary discursive signifiers that not only constrain our freedoms but infuse our very attempts to exercise it.

Moreover, in light of a technology that is allegedly both liberating and empowering in terms of Satoshi’s claim to have created ‘the absolute autonomy of money’, in assessing such promises, a Foucauldian approach impels us to move way beyond Kant’s [17] initial categories of negative restrictions and/or pathologies, to tease out what autonomy could mean in this regard, and how normative injunctions may inhere as much in overt institutional laws and practices as in discontinuous tropes and traces that make up discourse and propel us to avow certain values over others.

In other words, just as prisons, hospitals and various other institutions and normalising practices have been the focus of a great array of Foucauldian analysis, the technologies of diagnosing deviance in this way does not merely produce a particular type of docile inmate or patient subjectivity among such confines and specific populations in question. In addition, it is disciplinary towards the broader public in the sense of ushering in a normativizing and medicalising vocabulary that apart from generating various forms of delinquency and measures against it, also provides a powerful framework for what it means to be normal and sane. This is what Foucault ([7], p. 43) means when defining discourse as not only that which was “already said” as in the text itself, but also that which is “not-said” or “never-said” by which he means a form of silent potential that may become actual in the way we experience discourse.

This according to Foucault [8] means that power and knowledge are intimately connected, and connected precisely in the way that discourse produces certain outcomes or ‘truth effects’, by the way terms relate, silence or accentuate other terms in and through the functioning of the discourse. In taking this theoretical move from the earlier Foucauldian (cf. [7]) writings on *The Order of Things*, to later works such as *Discipline and Punish* (1980) to its logical conclusion, provides us with a rather different understanding in which the autonomy of individuals as understood by Kant is largely undermined. Indeed, this is where Foucault [7] shifts the idea of agency away from the individual and from an archaeological reading of so-called *epistemes* in terms used, rather accentuating the relational nature of power through a more genealogical understanding of discourses that generate their own form of agency (cf. [4, 31]).

## Design and Methodology

As discussed in our introduction, the philosophy of money and its historical and cultural importance is of great interest for this project. Moving forward, the digitisation of this traditional system of value exchange poses new risks and opportunities that re-challenge the existing socio-economic structures of our society. The defined purpose of this paper is to explore the ethics of virtual currencies in Sweden and Japan respectively, to understand commonalities and differences between cultural attitudes to utility, governance, and monetary philosophy, as provided through the evolution of discourse. Let us begin then, with a brief discussion of the logic behind such an analysis.

### Discourse Analysis

The essential assumption of discourse analysis, of course, is that all objects and actions are phenomenologically perceivable, attached to a symbolic structure of signification and that their meaning is dependent on historically specific systems of rules and social factors [7, 10, 18, 19]. In this structure of signification, e.g. semantics has a prominent role because of the consensual meaning symbolised through spoken language. Discourse is, thus, the realm of symbolic meaning in which social practices construct and contest conceptions that constitute social reality [18].

Discourse theory goes beyond the positivistic and naturalistic notions of knowledge and method, challenging scientific laws on social reality grounded on empirical generalisations . Furthermore, argues Laclau  [18], discourse theory does not surrender to naïve conceptions of truth and in contrast bases validity on the adequacy in socially constructed identities conferred on social agents. For everything that is written or spoken, there is at the same time a silent discourse of potentials that is never manifested for various reasons, and the discourse that we experience is the result of some potentials becoming actualities [7].

Moreover, discourse analysis is well suited for analysing policy discourse on social media platforms. In her paper, Sam [27] seeks to capture how localised narratives influence public opinion, and offers Foucauldian discourse analysis as a suitable qualitative method to explore how these narratives unfold. What is particularly useful about the approach for the purposes of this paper, is the linkages to policy development. Since cryptocurrency is a relatively new technology, the governmentality of it is very much an underdeveloped area. Comparing the two nations’ approaches to regulation can reveal interesting insights into how philosophical differences translate, from user attitudes and behaviours, into policy.

For this piece of technology research, capturing the discourses that inform people’s understanding of how a technology works, therefore, its utility and why it exists, provides us with a unique perspective on the adoption process. In understanding the cultural attitudes toward cryptocurrencies, we can begin to unravel the motivations behind a given technology’s use, and then compare these attitudes between cultures. Whilst this might be interesting from an innovation perspective, how these hopes and dreams or fears and hesitations of users and developers, transition into policy in each nation will also give us a unique view of the ethical considerations at multiple levels of society. Ultimately we aim to understand, why are people discussing virtual currencies, and in what context?

The internet, and social media in particular, is an increasingly important conduit where people express themselves openly. It allows them to communicate without fear of reprisal since they can post anonymously, and with relative ease [21]. Therefore, it also offers access to demographics that are difficult to study with traditional methods, such as those who use cryptocurrency for illicit activities. Social media also has its own rules of discourse, shaped in large part by the medium (cf. [22]). On Twitter, the medium takes place in different forms such as a tweet, retweet, or subtweet. Each of these activities is also laden with meaning not explicit within the text provided. These posts become a matter of record, which are easily retrievable through automated means, and their contents are rich with qualitative data such as text, images, photographs, and videos.

Thus, to understand more about the context and discourse of virtual currencies in both countries, the team decided to begin by analysing user attitudes from social media. In their paper, De Simoni et al. [5] explore how internet forums are a key source of hard-to-reach groups, and use discourse analysis to analyse and understand stroke patients’ issues and behaviours “with a view of better informing healthcare interventions and policies”. In particular, they use a frequency count of individual words as a useful submethod to highlight how important certain concepts are to people. Co-location of common concepts then allows researchers to infer correlations between concepts and their significance, which can be verified manually using other qualitative means. Baker [1] for example, uses critical discourse analysis in his review of UK media bias, and plots how the different types of words are used linguistically in combination with one another. This submethod has the main advantage of drawing objectively relevant results from the corpus, rather than searching for specific terms.

In general, discourse analysis on large volumes of data is a lengthy process, especially if done manually. For the purposes of this paper, the data collection and downselection is automated using python (and corresponding data science libraries), and key concepts and topics that are more heavily discussed are highlighted through topic modelling. Once identified, we then investigate concepts that are interesting from an ethical perspective, and focus our resources on understanding the context and nuance around them. First, however, it can be useful to understand a little of the theory behind topic modelling to understand how this process identifies dominant topics among the noise.

### Topic Modelling

Discourse analysis is often criticised for its subjective methodology [2, 25], hence, we have searched for a more scientifically rigorous approach that can help us objectively digest all that we have collected. Jacobs and Tschötschelb [14], and Törnberg and Törnberg [29] both demonstrate how topic modelling can be used to analyse social media discourse, and describe it as a method that inductively finds recurring clusters of co-occurring words in a text, without using pre-set keywords.

The algorithm behind topic modelling is called Latent Dirichlet Allocation [3], in which corpora (bodies of text) are converted into statistical representations of their component word distribution. Those wishing to understand the complexities of this algorithm are recommended to read the original paper, or more recent reviews such as Jelodar et al. [15], who explore the methodology in more technical detail.

‘Topics’ that are ‘discovered’ by this method of analysis are systematically produced from the text itself, rather than the researchers’ own interpretation, and it relies on the linguistic assumption that words that are co-located within a given text, tend to have relational context. It is important to note, however, that even if these recurring clusters are procedurally generated, the interpretation of what they mean and how they relate to one another is still subjectively constructed by the analyst. Hence, topic modelling is rather a useful tool to aid and expedite the discourse analysis [14].

### Data Collection and Selection

To standardise our data collection process for this study, we used an online media monitoring platform called ‘Notified’. The Notified platform is connected to various internet-based media sources, and gathers posts in real time for later analysis. It collects mainly from social media platforms such as Twitter, Instagram, Facebook, and YouTube, whilst also collecting data from forums, news articles, and blog posts. The platform was chosen, first due to one of the researcher’s personal connections which allowed our team to access a certain amount of data streaming free of charge, but also because it automates the data collection process.

To filter relevant data for this study and to avoid exceeding our data subscription limitations, Notified was set up to track posts that had any of the Swedish or Japanese key words crypto (krypto), cryptocurrency (kryptovaluta, 暗号通貨) or 仮想通貨 (virtual currency) within them. In addition, we included some systemic constraints to avoid including excessive amounts of spam posts which would contaminate our dataset. To maximise the amount of meaningful data we could collect, the platform was left scraping posts for a period of 9 months, between October 3rd 2019, and August 3rd 2020. Although older posts exist that could be retroactively scraped by other third-party software, the purpose of our study is to compare discourses between nations, hence, this is easier if the data are gathered across the same time period.

**Fig. 1:**
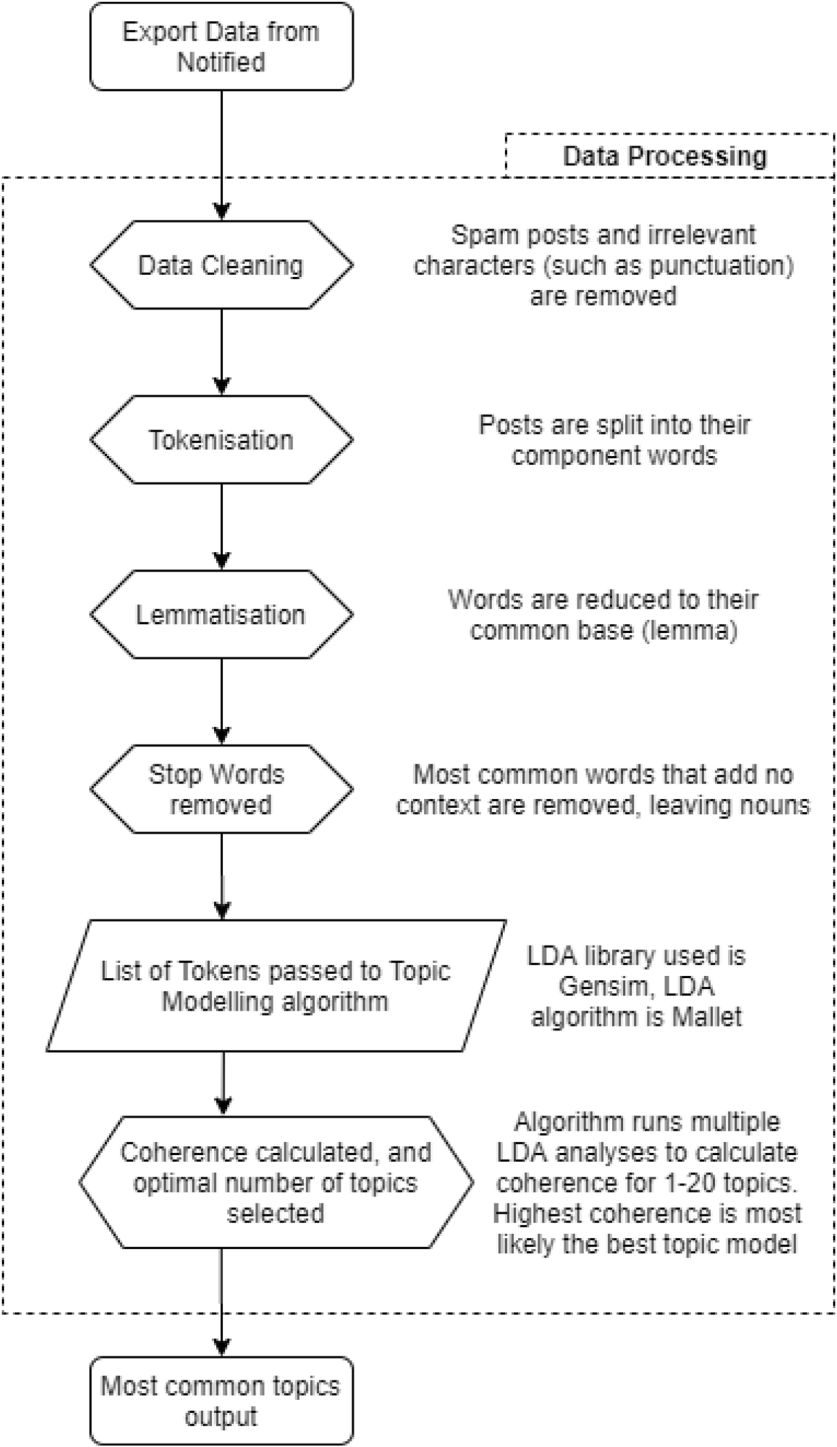
Main steps for data processing

Prior to LDA analysis and topic modelling, the data must be standardised and formatted appropriately to align with the input requirements of the model. The process is relatively straightforward, but requires some explanation for both Swedish (SV) and Japanese (JP) datasets. The main steps taken for this process are highlighted in Fig. 1, and described as follows.

First, data captured by Notified between the observation window (03/10/2019–03/08/2020) are exported to a Microsoft Excel file (a limitation of the platform).

These data are then pre-processed in Python using an open source package called Pandas, which constructs a data frame (rows and columns) based on the exported data. We then procedurally remove spam, special characters and letter combinations (such as @RT or HTTPS), and irrelevant data that would otherwise contaminate the analysis, from each cell in the data frame.

Using the package NLTK (Natural Language Toolkit) and Janome (a Python library for Japanese text analysis), each post or Tweet is tokenised, which essentially separates each word into its own independent unit within the data frame. This allows us to construct bigrams or trigrams and count the frequency of co-occurring words, which is important for the LDA analysis when using Japanese characters.

Following tokenisation, a lemmatisation algorithm is used to reduce each word to its base; words that are used more frequently, but in different tenses, are then counted together. For example, in English, ‘talked’, ‘talking’, ‘talker’ all reduce to the verb ‘talk’. This can sometimes be problematic if a word is contained within another word, however, such as ‘stalked’. Hence, it is necessary to follow a collection of rules which govern the lemmatisation process, known as a ‘model’. We had intended to use the SpaCy library for both the Swedish and Japanese datasets, since the package has models trained on social media posts, however, a Swedish language model was not available at the time of writing. Consequently, we used the Stanza package which is trained on the Talbanken treebank (SBX). This lemmatisation step is perhaps not strictly necessary for the Swedish data, but is useful for adjusting the output if we, for example, wanted to analyse sentiment at a later date. In the case of the Japanese data, Janome was used for lemmatisation.

Before running the LDA analysis, we remove all stopwords from our tokens; common words such as ‘is’, ‘a’, and ‘to’ which do not provide any context and would otherwise contaminate the results. For our purposes, we return a list of nouns for each post or Tweet, and then pass these lists into the Gensim library, using the Mallet LDA implementation algorithm to analyse the distribution of words.

The output is a collection of keywords, organised by strength and similarity of semantic structure; how strongly these ‘topics’ correlate to the data is indicated by a coherence score. This method, therefore, acts as a preliminary filter for our data, presenting us with a condensed set of potential—but not explicit—‘themes’ that can be used to guide our exploration of the various discourses contained within. We run the algorithm 20 times to find the number of topics with the highest coherence score, meaning that we select the ‘topics’ where the model is more confident that the quality of those topics matches the semantic structure.

Finally, we map these topics back to our original post or tweet data, and order them by those with the greatest contribution to the topics from the model. In this way, we systematically select data with the highest relevance for the purpose of our discourse analysis. The Notified platform also conveniently provides URL links to the source of the post or tweet, which can then be used to follow the evolution and context of discourse, throughout the entire thread.

There are, of course, by the very nature of comparing different cultures and language, differences to consider when designing and running such automated processes. We looked for the richest and most diverse data sources that Notified could provide; for Sweden, this was online forums such as Flashback. However, the platform was only capable of scraping Japanese data from Twitter. Using topic modelling with Twitter data presents some challenges due to characteristics such as the short length of each tweet [12]. We addressed this by removing twitter posts that contained less than 2 tokens from analysis. Consequently, the volumes of data gathered by Notified were vastly different (SV—1762 posts versus 234,653 tweets—JP), most likely due to differences in population size (SV—10 m versus 126 m—JP), as well as the internet-using behaviour and culture of the inhabitants. We accept this as a limitation of the study but argue that since we are comparing the outcomes, provided we have enough volume to perform the discourse analysis, the results are still relevant and of interest.

It should also be mentioned, there are nuances in the way the language is processed by the relevant models. Since there was no SpaCy model for the Swedish data, we were forced to use the Stanza model, which is trained on more traditional Swedish. This may have had an impact on the coherence of the LDA analysis, as the input data was modern internet-based language. Future iterations of this study may revisit the analysis if and when a user-friendly social media trained model is available.

## Analysis and Results

### Sample Characteristics

As mentioned previously, for the Japanese data, the Notified tool returned posts from Twitter. An initial examination of the data indicated that there were a high number of spam posts, even with the constraints we had used to filter the search. To clean up the data, we identified posts with identical content and posts with a user name clearly indicating a bot account, and eliminated them. In addition, we limited the data to those posts which had at least one Like, Retweet or Reply to further reduce the amount of spam in the data [16].

After cleanup, the Japanese data consisted of 77,527 posts from 34,203 unique user accounts. The median number of posts was one. Approximately a third of users in the data (32%) posted only once about the topic. The accounts with the highest number of posts corresponded logically to accounts dedicated to virtual currency/cryptocurrency topics. The maximum number of posts by one user was 298, from an account that focused on virtual currencies. The Japanese corpus consisted of 1,679,229 tokens.

For the Swedish data, the Notified tool returned posts from several online forums, the largest of which was Flashback. This forum is well known in Swedish culture as an upholder of freedom of speech, and seeks to “continue to offer a free social debate, without external influence, which is open and accessible to all” [6]. One might consider the demographic of such a forum relatively niche, and libertarian when compared with Twitter, however, according to the recent Swedish Internet survey [13], 32% of the internet-using population have used Flashback (though only 1% use it daily), versus 24% for Twitter (7% daily). In addition to Twitter’s lower adoption and 144 character limit, we argue that, for the Swedish context, the Flashback platform provides a richer and broader look at what individual users are discussing in relation to our search terms. Many of the threads on this forum liberally discuss topics that sit outside of accepted social norms, such as drug use, prostitution, and hacking. Hence, users of this forum obviously value concepts such as anonymity and autonomy. For this reason, it is a good resource to uncover ethical discourses for our target line of inquiry, though we accept that it captures the opinions of a particular demographic.

Unfortunately, the Notified platform also captured posts that did not link to valid threads. Some of these captured posts were duplicates or lost completely due to the format of the source website. Where this has occurred, the extraneous posts have been deleted. Hence, after initial data cleansing, the number of posts was reduced from 1905 to 1762. The average length of a post was 70 words, or 415 characters, from 672 unique authors. After lemmatisation, the Swedish corpus consisted of 39,147 tokens.

### Visualisations of Results

**Fig. 2:**
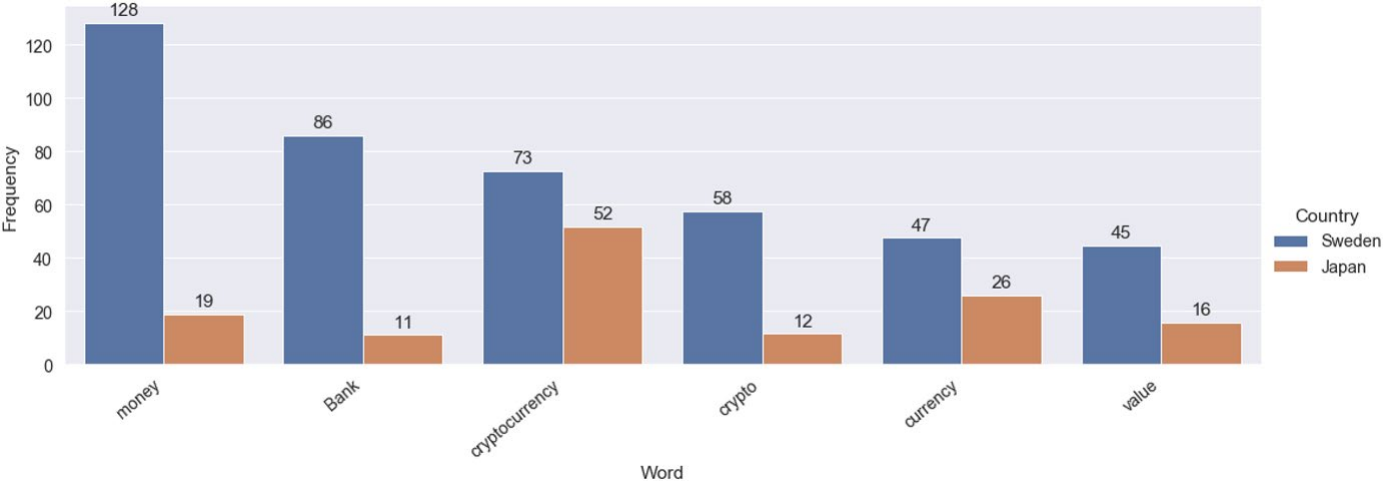
Most frequent words that appear in both the Japanese and Swedish data

Figure 2 shows the most frequent words for both countries that appear together, when translated to English. What is interesting to note about these results are the differences and similarities in relative usage of particular terms. For example, Sweden has a much higher relative usage of the words ‘Money’, ‘Bank’, ‘Crypto’, ‘Currency’ and ‘Value’. Looking more broadly at the rest of the data, both Sweden and Japan have a similar relative usage of the word ‘Investment’. Whilst this is a reflection of the relative frequency of tokens occurring throughout the datasets, these differences and similarities may have underlying cultural causes which can be of interest to this study, and can thus, offer a potential direction to target our efforts for the discourse analysis. Does Sweden consider virtual currencies to offer a replacement to the traditional monetary systems, more so than Japan, for example?

#### Coherence Test

**Fig. 3:**
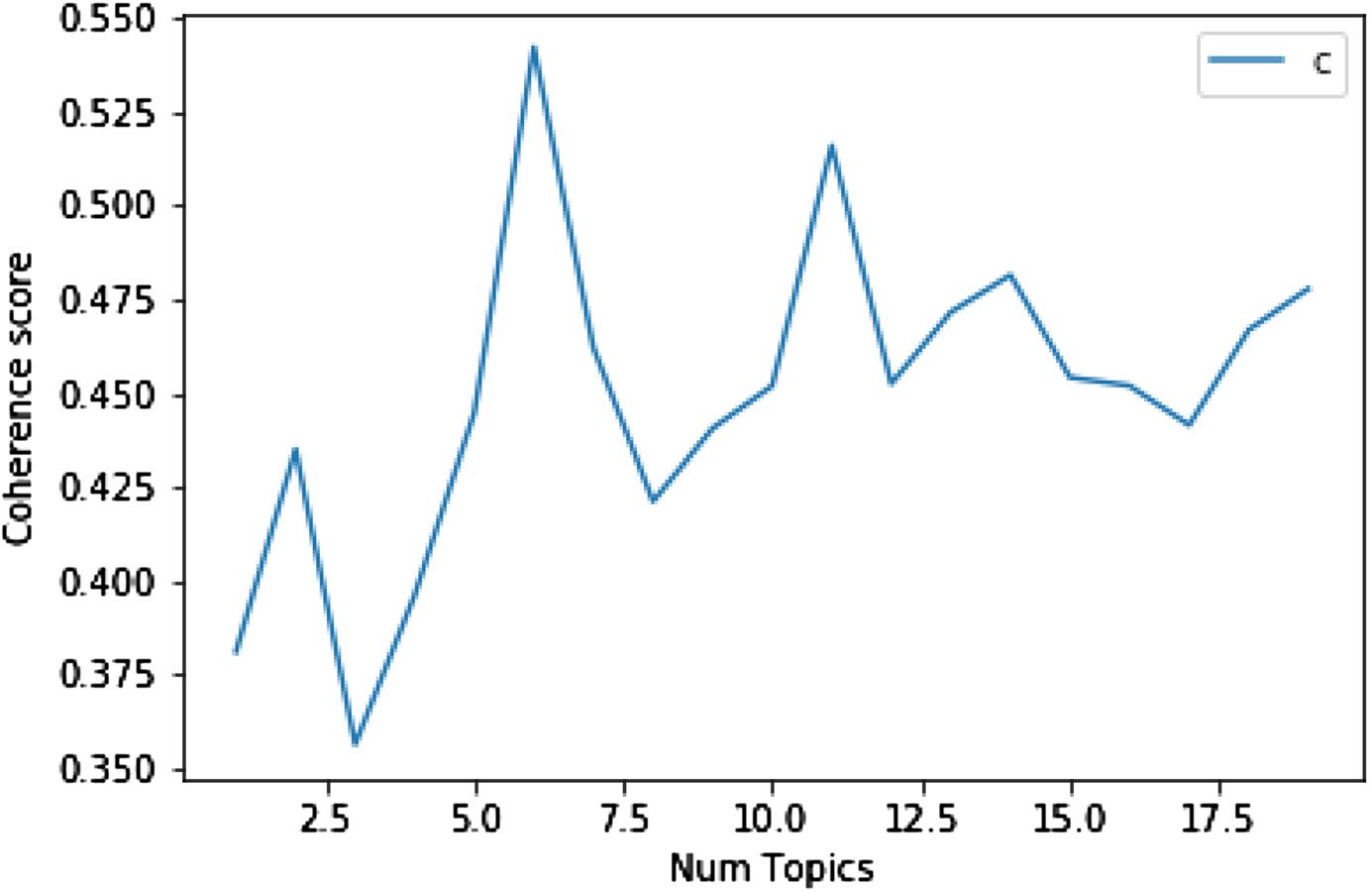
Swedish coherence score versus number of topics

Figure 3 shows the coherence scores for 1–20 topics for the Swedish data. For the Japanese data, the highest coherence was at 10 topics. For the Swedish data, the highest coherence was at 6 topics, but we see a second peak at 11 topics. These can be interpreted as sub topics which already fit into the main categories, but are useful to explore if we want to dive deeper into less common discourses.

#### Topic Network Maps

**Fig. 4:**
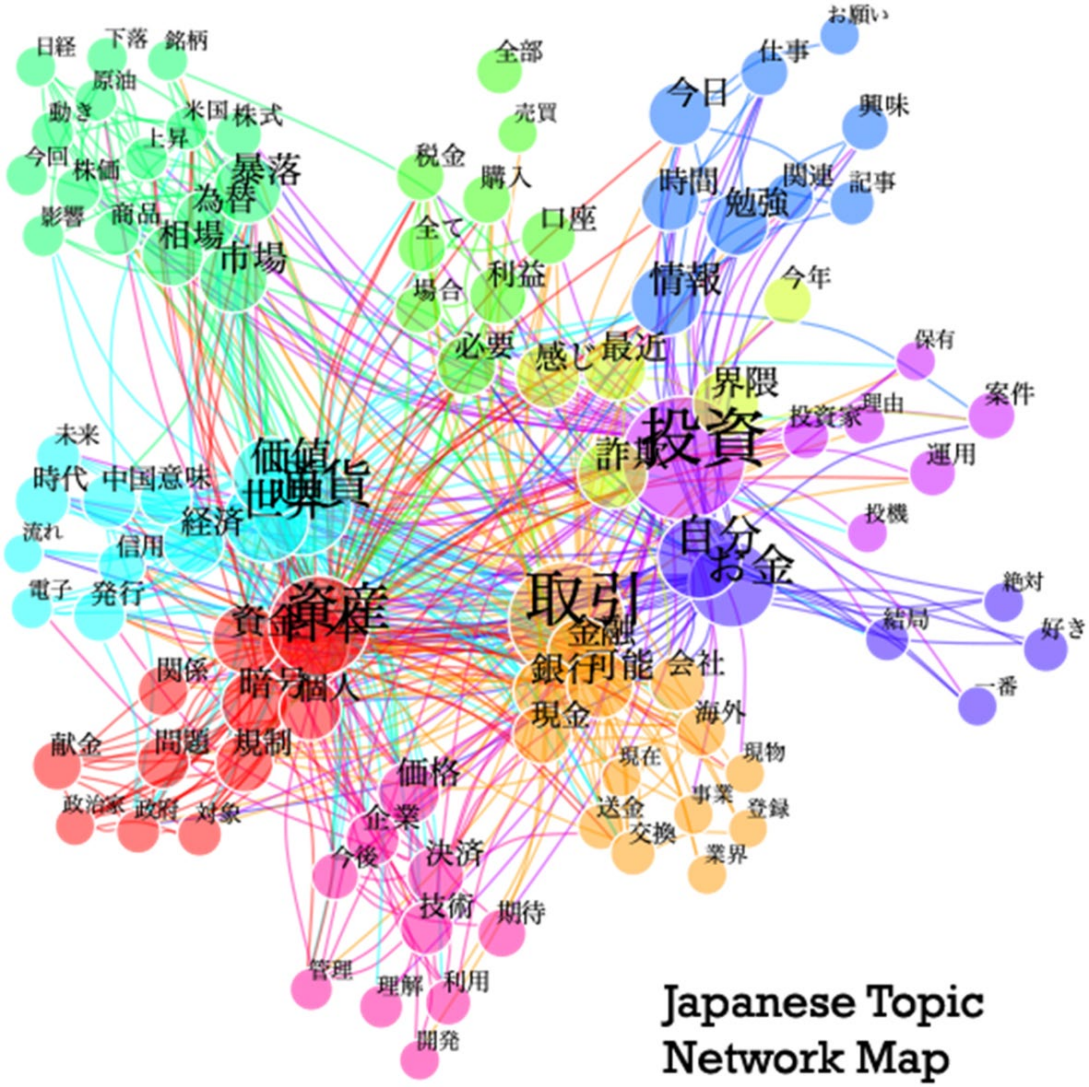
Japanese Topic Network Map

**Fig. 5:**
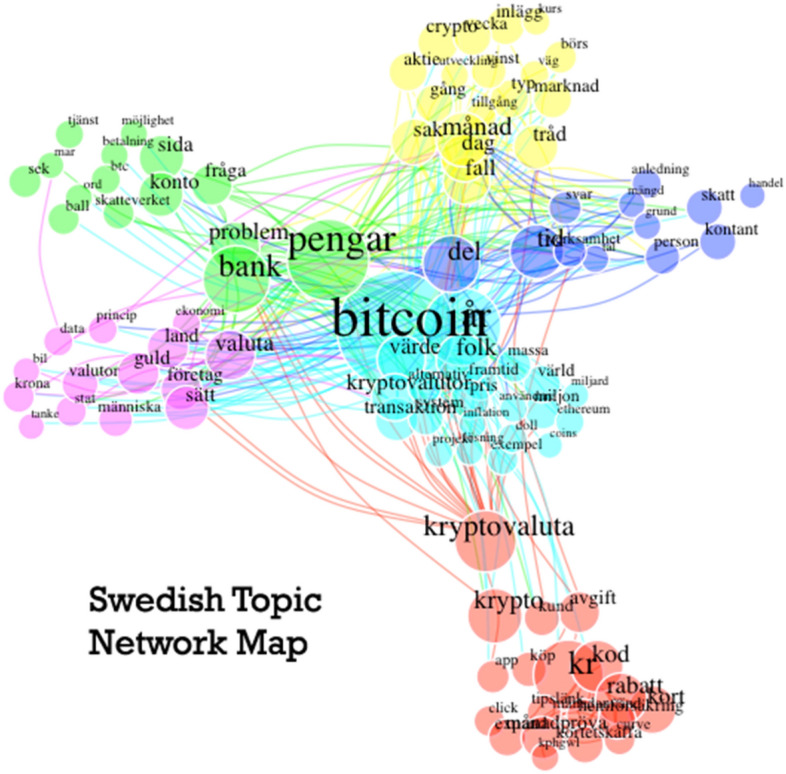
Swedish Topic Network Map

Figures 4 and 5 are topic network maps. These visualisations primarily show the clusters of keywords generated by the topic modelling algorithm, with each topic colour coded. They also demonstrate the interrelatedness of these words, by mapping which words are used alongside one another. The larger circles indicate a more commonly used word, and the lines between circles show which words are used together within the source data. In-line with the recommendations from Jacobs and Tschötschelb [14], we use these graphs to help interpret the topics and guide our discourse analysis.

The Japanese Topic Network Map reveals discourses that are different and are common to both Sweden and Japan. However, what is typical in Japan is that the analysis sheds light on the fact that the majority of the discourses surround words such as “investment, 投資”, “transaction, 取引”, “me or myself, 自分”, “capital, 資産”, “currency, 通貨” and “value, 価値”. In Japan, perhaps because of the government policy that is inclined to be more capitalist than socialist, there seems to be a trend in cryptocurrency-interested people to “invest into themselves, 自分投資”. After the 2008 Lehman Brothers shock, people tended to think that they could not rely on government policy, safety-nets and/or social welfares, but rather, need to help themselves out with their own effort. Therefore, one needs to invest in oneself, such as getting  a licence, learning new skills, and among them includes investment to cryptocurrency. Such discourses may indicate a hidden mistrust of the Japanese government by Japanese cryptocurrency market players.

Due to the high-volatility rate of the cryptocurrency market, Japanese users are well aware of the situation of both domestic and international markets, and oftentimes are using Twitter as a platform in sharing information regarding newly introduced domestic laws and policies, as well as what is going on in other commodity markets. In addition, what might be typical to Japanese discourse is that there is a cluster of discourse group that uses words such as “feeling, 感じ”, “like, 好き”, and “wishes, お願い” that indicates there are portions of people in the Japanese market who are amateurs, investing into cryptocurrency without much knowledge on how the market and its volatility may correspond to other commodities; they are investing based on their instinct, than on the market information. This indicates that there are both amateur and professional investors and traders participating in the Japanese cryptocurrency market, and that there might be a huge hidden divide between amateurs and professionals in terms of their motivation in participating and the reason for investing or holding cryptocurrency.

The Swedish topics highlight a strong focus on the main cryptocurrency, Bitcoin, but also the concepts of money, utility, assets, and markets. Many of the main key words are well connected to one another which suggests a strong link between topics. For example, ‘Valuta’ (currency), ‘pengar’ (money), ‘bank’, and ‘problem’ are all closely co-located with larger circles, which suggests that many posts discuss problems with the banking and monetary systems, perhaps exploring how cryptocurrencies may affect them. Other, less obvious words such as ‘guld’ (gold), and land, can be interpreted as a reference to the utility of cryptocurrencies, perhaps as an asset class or secure store of value. There are also references to the Swedish tax authority ‘skatteverket’, and taxes more generally ‘skatt’, which are linked to words such as ‘problem’, money (‘pengar’), and ‘bitcoin’. There are many ways to interpret these topical interrelations; it may suggest that users have a problem either with paying taxes on cryptocurrency earnings, or perhaps the discussion is about avoiding taxes entirely. To really understand what the context is, we must dive into the source data highlighted by these topical analyses, and begin our discourse analysis.

### Discourse Analysis of Social Media

In Japan as much as in Sweden, cryptocurrencies are broadly used and there are vibrant and ongoing debates on social media as to the significance of this new technology. By organising our data through the use of topic modelling, we pick up on some of the more salient discourses that have crystallised over time. We can see that investment opportunities, security issues and questions around anonymity are all prevalent concerns that emerge in ongoing discussions in both countries. Generally, there seems to be more homogeneity around the idea in Japan that the value of cryptocurrencies are essentially to be found in growth potential and investment opportunities.

Particularly in Japan, the majority of discourses surrounding cryptocurrencies are concerning the market price in relation to other markets such as stock and bond markets, as well as forward transaction of the commodities such as gold, silver, palladium, oil, soybeans, sugar, and wheat. There is also a group of discourses that surrounds uncertainties of the future cryptocurrency market, that includes discourses on technological issues such as quantum computing, quantum cryptography, and block chain security. Oftentimes the discourses show that people see a strong correlation between the crypto-technologies and block chain technologies; they assume the market volatility gets higher when there are articles published on such new computing technologies that could impact the stability or security of the market.

Discourses on uncertainty of the future also covers international issues such as China and the Chinese Digital Yuan, the U.S. and its policy to Facebook’s planned digital currency Libra (now rebranded to Diem), and Brexit and its impact on the international monetary market. The discourses that point out problems specific to Japanese markets are Japanese laws on taxation; that taxes higher to revenues earned from cryptocurrency trading (50% taxation) compared to the revenues earned from stock trading (21% taxation), and to a new amendment law that allows cryptocurrency to be used for donation to the politicians without any tax (Amendment to Political Funds Control Law, 政治資金規制法改正). However, as mentioned, the majority of the discourses show interest on growth potential and investment opportunities for the future price hike of the cryptocurrencies that leads to a continued interest into investments in cryptocurrencies. This, for instance, could be found in the following tweet:

“When I see that the cryptocurrency price fell down steeply after the announcement of the quantum computing technology development, I am convinced that the “money is actually based on the trust”. However, quantum computing technology requires certain more time to be practically applicable, so I think the price fall shows that the market responded to the news but the price itself will stabilise once the people in the market understands the fact that quantum computing technology is still not at the stage to be released soon.” (Japan).

Growth potential and investment opportunities are indeed also recurring themes in Sweden but, as Fig. 2 above indicated, with the significantly higher frequency of terms like ‘money’ and ‘bank’, we can start to appreciate a distinction even at this somewhat crude tip of the iceberg level, wherein the aggregated Swedish content seems on the whole to be more open to Bitcoin having value not just as an investment like any other, but as a form of money and alternative to banking matters. In Japan, there are a wider array of cryptocurrencies to invest in, thus, it seems that people are not only focusing on Bitcoins but also are committed to diversified investment of other cryptocurrencies such as MONERO, NEM (XYM), and ETH.

If we dig a little deeper into each identified topic to conduct our discourse analysis and more thoroughly figure out the dynamics through which these discourses play out, we quickly get into a multitude of streams and seemingly organic interactions that make up such overarching topics. For example, in both Sweden and in Japan, there are multiple discourses that point out the changing role of the conventional bank as a crediting, banking, exchange, and remittance institution (money transfer); and there are a multitude of voices that question the role of the conventional bank and their method of profit making.

“We are facing a time of great transition. There will be no need for the conventional business such as banking, crediting, stock and bond house, and security finance companies. All these companies that assured our credibility in moving the money will be no use because of the cryptocurrencies and the backup of blockchain technology. This means that we are not going to be centrally governed by the third party and we can act more individualistically in the money market.” (Japan).

Furthermore, in these exchanges, we can follow various threads with alternative concerns, for example, the excessive and worrisome electricity consumption required for the Bitcoin blockchain. We found this user on the flashback forum expressing his doubts about the future of Bitcoin in this way:

“Bitcoin is such an inefficient way of storing transactions and it is certainly not sustainable in the long run. Bitcoin was a good investment for those who went in early, but today one will have a better chance of getting rich through investing in Pi (RG: a newer cryptocurrency project that aims to facilitate low electricity consumption mining via smartphones) than investing in Bitcoin.” (Sweden).

To which another user retorts in a somewhat patronising tone:

“Typical ignorance from someone who doesn’t understand how difficult it is to bootstrap a decentralized project or who is only out for the last pump-n-dump Ponzi scheme (which Pi is), that will enrich the founders and impoverish everybody else.” (Sweden).

Such discourses are also shared among the Japanese users as well:

“It is nice to see the cryptocurrency technology and its system being realised as full-use in our daily life. However, I am a bit worried knowing that all these technologies are relying on the use of the electricity itself. Of course I understand it is part of investment into the future, but am worried about the consumed electricity. If we are to think about sustainability and an ecological future, I think we need to find a way to invest into renewable energy through cryptocurrency as well.” (Japan).

Other discourses in Japan, since it is situated in the midst of the growing Asian economy, reference more concerns on efforts and growth of other countries such as China and Cambodia that have announced governmental strategies to introduce sovereign cryptocurrencies. In other words, decentralisation of the authority is welcomed, but there is little empathy to be seen for the impoverishment of other countries through cryptocurrencies. Rather, a sense of impending crisis of Japanese economy exists, in the midst of rapid strategy change in other Asian countries.

“I heard in Cambodia there are plans to circulate cryptocurrency that could be used for business settlement using blockchain technology. This indicates that Cambodia can settle their business without any time-lag, therefore reduces the exchange risks for the Cambodian businesses” (Japan).

“In China, there are more than 500 companies listed that claim to be working on blockchain technology. The Chinese government is also backing up those new start-up type businesses and are changing their laws to strengthen such new types of business. I feel sorry that Chinese people have to endure more strict regulation in trade of with such government enforcement to new type of business, but perhaps the situation is better compared to Japanese government who cannot even tell the relation of blockchain and decentralisation” (Japan).

The interesting thing about this exchange is that although it seems rather arbitrary and idiosyncratic, it actually illustrates a dynamic that is quite emblematic of how the discourses on the Swedish forums and Japanese tweets play out. Posts could have raised a number of inherent or potential problems with Bitcoin and other cryptocurrencies, as they did in terms of its excessive electricity consumption, or about it not being such an attractive investment opportunity any more. It could have, as others do, raised concerns about cryptocurrencies facilitating dealings in narcotics, other illicit goods, money laundering or terrorism. Or for that matter, as another Swedish user expressed, about the role Bitcoin played in the funding schemes of the Russian intelligence unit GRU, to weaponize data in the Facebook disinformation campaign that purportedly interfered in the US 2016 election to get Trump elected. We find that for the most part, whatever the issues that users raise in terms of ethical concerns, the various forms of responses that tend to come back are somewhat homogeneous in both structure and content.

There are, however, some disparities between ideological discussions. For example, one Swedish user criticises Bitcoin, by virtue of the fact that Satoshi went underground as soon as the technology had been launched, and that this undermines Bitcoin’s credibility and constitutes a problem of accountability. To which another user retorts:

“Not really, …that is incorrect. What Satoshi was trying to counteract with Bitcoin, is the centralization of money via banks, and inflation, which in fact has been a great success” (Sweden).

In Japan, however, there are tweets that reveal that Satoshi’s ideology of decentralisation is not that well known compared to what appears in Swedish social media discourse, and this could be because of the nature of the demographics behind the datasets.

Along with the previous Japanese tweet concerned with the government’s slow responses to cryptocurrencies, there are also discussions on whether cryptocurrencies should be centrally governed by the nation state or should be kept private and decentralised, as seen by the following exchange.

“I doubt a bit when sovereign currency is going to use blockchain and call it as decentralised money, but perhaps the governments can at least detect money laundering far more easily by using blockchain” (Japan).

“What happens if the local governments are given rights to have their own local cryptocurrencies in Japan? I am quite sure that many local governments will be financially corrupt but that may actually show how people trust in the local economy as well. Perhaps it could be a good way to bring out the hidden issues of the local government in Japan” (Japan).

Much like what we saw in the earlier Swedish retort, there is often an element of annoyance over the fact that Bitcoin and other cryptocurrencies are questioned that may or may not come through in a patronising tone. There is also a reminder of the fact that decentralisation is a rather difficult affair, yet implicitly worth whatever issues the response aims to neutralise in this context. The fact that there is an implied value to such terms here is significant, because unlike the more explicit ethical terms associated with the voiced concerns ranging from electricity consumption to terrorism, it clearly illustrates the more silent dynamic in these emergent discourses whereby decentralisation has become associated with notions of empowerment, peer-to-peer, autonomy and freedom. Another user chimes in to support the formers retort by pointing out that Satoshi’s stepping down in fact strengthens the decentralised nature of the technology by virtue of it being leaderless:

“It is much more peer-to-peer as a result, and should instead be seen as a strength to credibility, rather than a weakness.” (Sweden).

The Japanese discourse resonates, with the hope of de-authorisation through decentralisation of money, and to regain the autonomy of money from authoritarian control.

“Cryptocurrencies work based on a fundamental thought of getting out of the control of the sovereign currency, but seeing the situation of Communist countries, I am getting a bit doubtful of the cryptocurrency system. I doubt if cryptocurrency could really work for the benefit of the people who seek for more freedom and liberty. Now, I am slowly getting to understand why Chinese people like to invest into gold and see gold as the best option for investment. Gold makes us ultimately anonymous anywhere in the world.” (Japan).

“Japan is going to face another down-shock of the economy in the near future. I can see that through Japanese slow decision making in politics and its trickle down effect to Japanese businesses. Soon the monetary field will be a three-cornered battle between the sovereign cryptocurrency, private-company issued cryptocurrency, and the original cryptocurrency with libetarian thought like Bitcoins…” (Japan).

However, what is different from Swedish discourses is that there is a gradual rise of skepticism of the use of blockchain technology in Japan. In other words, the underlining technology that enables the whole value proposition to some users of cryptocurrencies may, in reverse, hurt the ideology around autonomy and anti-authoritarianism, and strengthen the totalitarian and authoritarian aspects of money. This perhaps comes from the fact that the majority of the Japanese users are watching keenly on international trends not only libertarian countries such as the U.S. and Estonia, but also countries such as China and Cambodia that have a somewhat different agenda in regard of liberty and personal autonomy, and are more inclined to maintain totalitarian control.

## Discussion

In the wake of the financial crisis of 2008, we have seen austerity driven anger and disillusion, a rather cynical resurgence of protectionism, nationalism and xenophobia rippling through the global political landscape. As suggested here, paralleling these broader societal developments in response to the financial crisis we trace the initially rather peripheral innovation of Bitcoin that nevertheless aimed to revolutionise our dependence on banks through decentralisation. The blockchain technology that this new cryptocurrency brought to the fore had been devised to decentralise control in financial transactions and concomitantly provide an alternative as well as greater autonomy from banks and financial institutions.

In this view explicitly envisioned by the inventor Satoshi Nakamoto [23], blockchain technology extends the autonomy of money, which provides us with an underlying ethical question and vantage point from which much of our discussion will be borne out. Is it true, as suggested by its inventor, that blockchain offers us the ‘absolute autonomy of money’? It is certainly clear that in a technical and concrete manner, when sufficiently implemented, users are more directly able to use their funds without third-party control or consent that is required in other forms of digital payment methods. It is also clear from our empirical accounts that this increase in autonomy is something that appears to have more intrinsic value among Swedish users than among their Japanese counterparts. In Japan on the other hand, this ethical ideal or principle seems somewhat watered down and more of a means to an end in terms of powering the currency and facilitating investment prospects.

With increases in autonomy, there is also an increase in responsibility, more frequently picked up on by Japanese users. By virtue of not having someone ‘in charge’ there is of course no one to turn to and thus, no way to revoke or compensate faulty transactions, or to retrieve forgotten passwords for then irretrievable funds and fortunes. What is the value of absolute autonomy when there are obvious limitations to human reason, and what level of autonomy then might be most appropriate when we can be as negligent as we can be rational? Is it possible that it gives us too much autonomy?

As social beings, humans have obviously been confined by all kinds of tacit or explicit norms of what is proper economical behaviour for our particular station in life, that has obviously accompanied all kinds of societies from time immemorial to the present in a range of ways. For those reasons, it is not just the decentralisation aspect of this new technology that strengthens individual autonomy but also a greater degree of anonymity. In other words, it is clear that this innovation, if sufficiently implemented, enables us to more exclusively decide on how we want to dispense of this impersonal power that is money. In this sense, Satoshi’s words are quite true, Bitcoin strengthens our individual autonomy. The question is just, at what cost? Could we imagine that a certain amount of coercion effectuated by the awareness of passive to active surveillance of transactions, and the mere sense that we can be monitored or held accountable in one form or other is warranted, by better estimating our credit worth for loans or business dealings, or deterring us to spend our money in unethical or illegal ways?

This question raises the equally timely and timeless ethical problem around paternalism and autonomy that Satoshi assumed a particular stance towards, in both deed and word, but that one could equally find compelling arguments to assume the opposing view for. Should our freedom and autonomy be as absolute as possible, even if it means acting unethically or even against our interests, as we may not always know the course of action that would most readily attain them?

In contrast, certain well-placed paternalist guard rails might well be considered warranted if they managed to provide some guidance in how we are best to pursue those interests. However, where do we draw the line as to when these guard rails are merely optimising a means to an end, or coming to shape the very ends we aspire to pursue? In extrapolating from the broad array of social media posts under investigation here, it seems the Japanese users are quite pragmatic and would be more inclined to accept such guard rails in particular instances when the outcome might be beneficial to the subject, but yet express concern about the control becoming excessive. The Swedes on the other hand seem more attuned to the ideological aspects of the currency and the more principled connotations of Satoshi’s claim, in facilitating the absolute autonomy of money.

The question is perhaps most pointedly brought to a head by broadening the scope beyond monetary transactions and considering Google co-founder and previous CEO Larry Page’s position on the ultimate ambition of the search engine algorithm being to achieve better knowledge of what it is that someone wants, and suggest it, before the subject is even aware of what it is he or she wants [9]. Certainly in grappling with the cutting edge possibilities of AI in search engine optimization, this is fairly uncontroversial in a practical sense contemplating the suitability of guard-rails against imperfect information. However, with a somewhat more sociological and ethical perspective on the impressionable nature of the mind and bearing in mind the knowledge/power dichotomy put forth by Foucault [8], it poses a more fundamental question about freedom and autonomy, and could be perceived as alienating as it could be helpful to expose ourselves to such pre-emptive forms of ‘assistance’.

In the *Age of Surveillance Capitalism*, Zuboff [32] explains just how profitable such ‘assistance’ has become, as mining the behavioural surplus of our online activities for predictive purposes, is swiftly replacing oil as the number one commodity powering the global economy. We leave to the reader to determine whether there is an irony in putting forward these critical points about mining behavioural surplus, when this is precisely what we ourselves have been doing methodologically in this study by way of discerning attitudes from social media towards cryptocurrencies. However, in our defence, we have done so for scientific purposes as opposed to commercial ones, and concomitantly to reveal these inner workings for the sake of knowledge rather than quietly harnessing it for monetary gain. Secondly, as our inquiry specifically endeavours to explore attitudes towards not just cryptocurrencies but blockchain technology more broadly, we are hoping to foreground a more informed discussion about this technology and what regaining autonomy in one form or other could mean, whether against financial institutions or the behavioural surplus-predictive complex of the likes of Google and Facebook that epitomise the logic of surveillance capitalism.

## Conclusion

In this paper, we have demonstrated that autonomy as well as money are in a number of ways intimately connected. From the clay inscriptions of Babylon to coined money in Lydia and the ancient Greek city states and beyond, monetary innovation has in many ways reflected as well as been a harbinger of developments furthering personal freedoms and autonomy throughout much of our history. Just like coins enabled for instance the ancient Greeks greater autonomy over their material conditions and the ability to trade their way out of poverty, so too blockchain powered cryptocurrencies may be an option for among others the two billion unbanked poor of the world to become part of the financial system. These people are currently excluded from any banking system on account of bureaucratic practices that come with centralization, and the decentralisation of autonomy brought forth by the blockchain offers an opportunity to overcome such precarious conditions.

Money is essentially a form of impersonal power that may command the labour of others and the way we can exercise this power will undoubtedly have a profound impact on our autonomy, yet some cultures may cherish this autonomy more than others depending on a range of factors including trust in public and private institutions and ultimately in one another.

From our discourse analysis of social media posts about cryptocurrencies, we have been able to discern some similar and distinguishing characteristics and tendencies between Japan and Sweden. Whereas both countries seem to have a rather high degree of trust in public and private institutions, we have been able to detect certain nuances around what autonomy might mean in each respective cultural context and the extent to which this new technology in so far as it bears a significant impact on our autonomy is valued as an ethical and/or ideological ideal. Swedes in this regard tend to attribute a higher degree of intrinsic worth to the principle of autonomy through the lens of blockchain technology as opposed to the Japanese who seem to have a more pragmatic view of the role it may play as evidenced by these online forums.

What makes money such a fascinating object of study in this regard is that in a very real sense, it is virtual long before the ascent of virtual currencies such as cryptocurrencies, precisely because money is continuously confirmed as performatively valuable as long and only as long as it is put to use and treated as valuable. In this sense, the object of study is quite malleable and possesses both idealised and pragmatic qualities in its very nature, and why it has been so interesting to see how these inherent characteristics have been reflected in various discourses that accentuate certain cultural nuances from one culture to the next pertaining to such a profound ethical question around autonomy.

More generally, however, and considering there are clear resonances across these cultures, contexts and platforms, it is clear that the internet has narrowed the intellectual distance between nations and their inhabitants. Hence, what makes this study so exciting is the relative ease at which we can observe the formation and evolution of discourses across both societies, as well as the impact that cryptocurrencies are having, in real time. Whether the conversation focuses on the role of blockchain technology in climate change, politicians’ tax evasion, or the legitimate use of cryptocurrencies; what we see is essentially what Foucault [7] would consider the interrelational nature of disciplinary power playing itself out on an entirely new medium in real-time. To what extent the old institutional power of centralized authority will come to dominate these new domains, is still not clear. Nor is the precise extent of autonomy afforded by blockchain technology ever settled therein, as long as information remains free and the last word is rightfully in the next post of this ever-evolving discourse.
